# Targeting EGFR in Combination with Nutritional Supplements on Antitumor Efficacy in a Lung Cancer Mouse Model

**DOI:** 10.3390/md20120751

**Published:** 2022-11-29

**Authors:** Chih-Hung Guo, Wen-Chin Li, Chia-Lin Peng, Pei-Chung Chen, Shih-Yu Lee, Simon Hsia

**Affiliations:** 1 Micronutrition and Biomedical Nutrition Laboratories, Institute of Biomedical Nutrition, Hung-Kuang University, Taichung 433, Taiwan; 2 Taiwan Nutraceutical Association, Taipei 105, Taiwan; 3 Biotechnology, Health, and Innovation Research Center, Hung-Kuang University, Taichung 433, Taiwan

**Keywords:** anti-tumor signaling pathway, gefitinib, erlotinib, Lewis lung carcinoma, mice, selenium, fish oil

## Abstract

Selenium (Se) and fish oil (FO) exert anti-epidermal growth factor receptor (EGFR) action on tumors. This study aimed to compare the anti-cancer efficacy of EGFR inhibitors (gefitinib and erlotinib) alone and in combination with nutritional supplements of Se/FO in treating lung cancer. Lewis LLC1 tumor-bearing mice were treated with a vehicle or Se/FO, gefitinib or gefitinib plus Se/FO, and erlotinib or erlotinib plus Se/FO. The tumors were assessed for mRNA and protein expressions of relevant signaling molecules. Untreated tumor-bearing mice had the lowest body weight and highest tumor weight and volume of all the mice. Mice receiving the combination treatment with Se/FO and gefitinib or erlotinib had a lower tumor volume and weight and fewer metastases than did those treated with gefitinib or erlotinib alone. The combination treatment exhibited greater alterations in receptor signaling molecules (lower EGFR/TGF-β/TβR/AXL/Wnt3a/Wnt5a/FZD7/β-catenin; higher GSK-3β) and immune checkpoint molecules (lower PD-1/PD-L1/CD80/CTLA-4/IL-6; higher NKp46/CD16/CD28/IL-2). These mouse tumors also had lower angiogenesis, cancer stemness, epithelial to mesenchymal transitions, metastases, and proliferation of Ki-67, as well as higher cell cycle arrest and apoptosis. These preliminary results showed the Se/FO treatment enhanced the therapeutic efficacies of gefitinib and erlotinib via modulating multiple signaling pathways in an LLC1-bearing mouse model.

## 1. Introduction

Lung cancer is a major cause of cancer-related deaths worldwide, with non-small-cell lung cancer (NSCLC) accounting for about 85% of lung cancer deaths. Patients with NSCLC have a five-year survival rate below 15%, owing to a lack of early detection, a high recurrence rate, and the limited efficacy of anticancer agents. Therefore, there is a critical need to develop new treatments, including neoadjuvant therapeutic strategies [1].

The aberrant upregulation of the epidermal growth factor receptor (EGFR) and other receptor tyrosine kinases, such as the transforming growth factor beta receptor (TβR) and AXL, in NSCLC tumors is associated with poor outcomes; meanwhile, their overexpression activates the downstream PI3K/Akt/mTOR, Raf/MEK/ERK, and JAK/STAT pro-oncogenic signaling pathways, resulting in tumor proliferation, angiogenesis, migration and invasion, and anti-apoptosis [2,3,4]. The binding of transforming growth factor-β to its receptor TβR can activate the EGFR, angiogenesis, and the epithelial–mesenchymal transition (EMT) in cancer cells [5,6]. The overexpression of AXL and its ligand Gas6 is also associated with EGFR activation and acquired resistance to the EGFR tyrosine kinase inhibitor (EGFR-TKI) in EGFR-mutated NSCLC cells [7]. β-catenin is a pivotal mediator of Wnt signaling and the aberrant activation of Wnt/β-catenin signaling is found in gefitinib/erlotinib-resistant NSCLC cells [8]. The crosstalk between the EGFR and Wnt/β-catenin can contribute to the invasion and metastasis of NSCLC cells [9]. AXL degradation or the suppression of the Wnt/β-catenin pathway, in contrast, may improve patients’ responses to anticancer drugs and decrease EMT marker levels [10,11]. Furthermore, the crosstalk between the EGFR and programmed cell death protein 1/programmed cell death ligand 1 (PD-1/PD-L1) is observed in the NSCLC, which downregulates T cell signaling and helps the tumor evade immune surveillance [12]. Thus, the effective modulation of multiple cancer signaling pathways is important in NSCLC treatment.

Recent studies have demonstrated that an essential micronutrient, selenium (Se), and n-3 polyunsaturated fatty-acid-enriched fish oil (FO) have well-established anti-cancer properties among different tumor types through various signaling pathways in the tumor microenvironment and in immune cells [13,14,15]. For example, the chemotherapy agent doxorubicin, in combination with Se, markedly decreased the proliferation, migration, and invasion, and increased the apoptosis of the EGFR and KRAS-activating mutant A549 lung adenocarcinoma cells as compared to doxorubicin alone [16]. The combination of Se and FO significantly decreased the growth and promoted the apoptosis of A549 cells as well as induced AMP-activated kinase (AMPK) activation and β-catenin downregulation [17]. The combination treatment of Se and FO induced the apoptosis of cancer stem cell-like A549 sphere cells, further decreasing the cisplatin resistance [18]. This combined treatment also decreased the AXL levels and gefitinib resistance in EGFR-mutant HCC827 lung adenocarcinoma cells, thereby increasing apoptosis, suppressing the EMT, and eliminating cancer-cell stemness [19]. Therefore, hypothetically a combination of Se plus FO can be a potential adjuvant therapy to increase the efficacy of anticancer agents in NSCLC.

The EGFR wild type and KRAS mutant Lewis lung carcinoma (LLC1)-bearing mouse is widely used as a model for testing the molecular mechanisms, anti-metastatic activity, and immunity of anticancer agents, although it is not an EGFR-mutant lung cancer model. Treatment with gefitinib or erlotinib, a first-generation EGFR-TKI, often used in NSCLC treatment, can cause lung metastasis inhibition and the inhibited phosphorylation of EGFR in LLC1 cells treated with radiotherapy and LLC1-bearing tumors [20,21,22]. Gefitinib treatment improved the progression-free survival ratio among patients with EGFR-mutated NSCLC but was not observed in those without the EGFR mutation [23]. Recent studies have shown that a combination of gefitinib and a commercial formula containing Se/FO/coenzyme Q10 plus multi-antioxidants markedly inhibits EMT markers’ expression over gefitinib alone via the suppression of TGF-β and hypoxia-inducible factor-1α (HIF-1α) expression [24]. Meanwhile, it is supposed that Se and FO are the critical components of the formula for anti-cancer efficacy. The commercial formula also enhanced the anticancer effects of radiotherapy by decreasing lung metastasis and EGFR expression and increasing apoptosis in LLC1 tumor-bearing mice [25,26]. On the other hand, a previous study has demonstrated that Se/FO can enhance the anticancer activity of bevacizumab by inhibiting tumor EGFR, TβR, and AXL proteins in breast tumor-bearing mice [27]. Thus, Se/FO may inhibit cancer activity, such as angiogenesis, EMT, metastasis, and anti-apoptosis via modulating the tumor receptor signaling molecules in NSCLC cells. In the present study, it is interesting whether combining first-generation EGFR-TKI with Se/FO increases the in vivo anti-cancer efficacy over that of EGFR-TKI alone in NSCLC without the EGFR mutation, although the KRAS mutation inhibitor is currently available. 

This preliminary study aimed to address the anticancer efficacy of combination treatment using gefitinib or erlotinib with nutritional supplements (Se/FO) in an LLC1 lung carcinoma cancer model. The selective targeting of oncogenic signaling molecules and the tumor immune microenvironment molecules (i.e., PD1/PD-L1/cytotoxic T lymphocyte associated antigen-4, CTLA-4/NKp-46/CD16/CD28/CD80/IL-2/IL-6) were evaluated. 

## 2. Results

### 2.1. Inhibition of LLC1 Lung Cells Growth by EGFR-TKI (Gefitinib or Erlotinib) 

**Figure 1:**
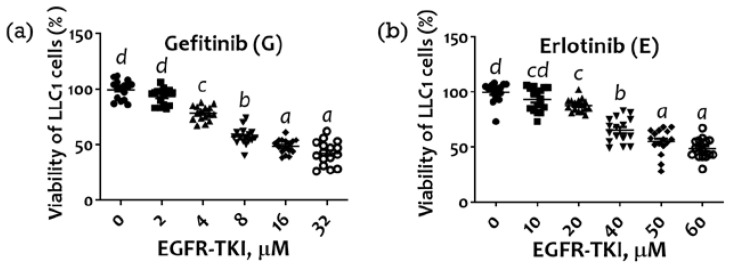
Inhibitory effect of EGFR-TKIs (**a**) gefitinib and (**b**) erlotinib on the LLC1 cell viability. Cell viability was analyzed by MTT assay. The IC_50_ values resulting in 50% cell growth inhibition via the 48 h treatment with gefitinib or erlotinib compared with untreated control cells were calculated. Means sharing the same superscript (a, b, c, d) are not significantly different from each other (*p* > 0.05); means with different superscripts are significantly different from each other (*p* < 0.05).

The median IC_50_ values for gefitinib and erlotinib in LLC1 cells are 4.79 μM and 40.96 μM, respectively. Compared with the untreated group, the treatment of LLC1 cells with gefitinib (at 4, 8, 16, and 32 μM) significantly showed growth inhibitory effects after 48 h of incubation (*p* < 0.05) (Figure 1a). Additionally, there was higher growth inhibition in all groups treated with erlotinib (at 20, 40, 50, and 60 μM) than that of the untreated group (Figure 1b).

### 2.2. Effects of Combination Treatment on Body Weight, Organ Weight, and Subcutaneous Tumor Size of LLC1 Tumor-Bearing Mice

**Figure 2:**
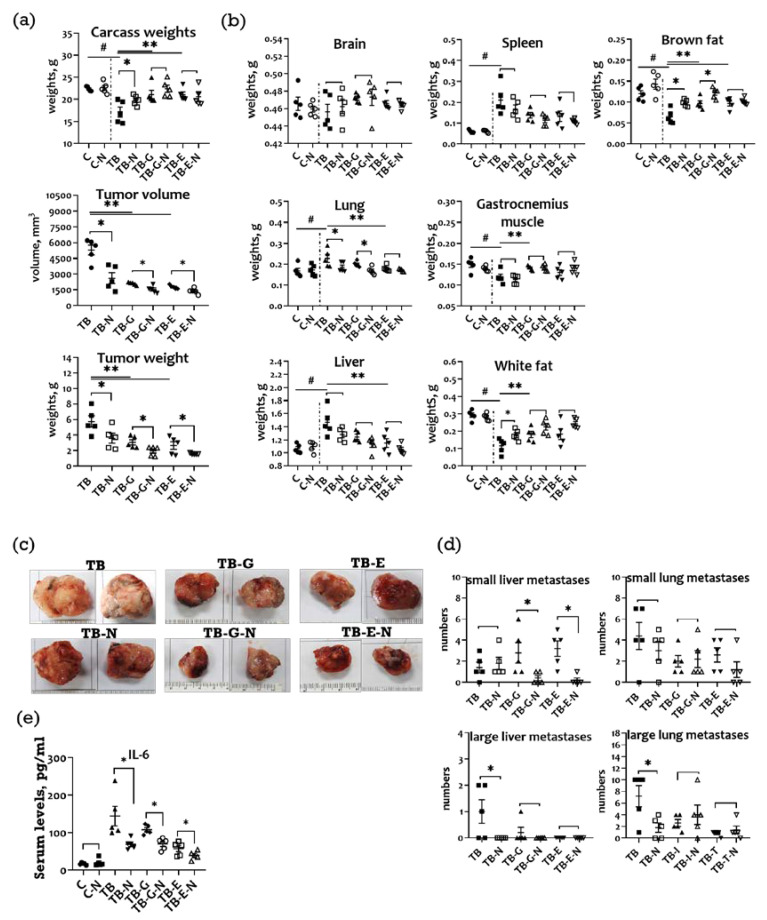
(**a**) Body weight, tumor weight, and size; (**b**) Weight of organs and adipose tissue; (**c**) Representative images of excised tumors; (**d**) Number of tumor nodules in the lung and liver tissues; and (**e**) Serum interleukin (IL)-6 levels of Lewis LLC1 tumor-bearing mice. The mean count of metastatic nodules in isolated tissues was determined by three laboratory technicians. Healthy mice were treated with vehicle (C) or FO/Se (C-N); LLC1 tumor-bearing mice were treated with vehicle (TB) or Se/FO (TB-N), gefitinib (TB-G) or gefitinib plus FO/Se (TB-G-N), and erlotinib (TB-E) or erlotinib plus Se/FO (TB-E-N). Data are expressed as the mean ± SEM (*n* = five mice in each group). * *p* < 0.05 TB vs. TB-N, TB-G vs. TB-G-N, TB-E vs. TB-E-N; ** *p* < 0.05 TB vs. TB-G or TB-E; # *p* < 0.05 TB vs. C.

Compared with the healthy controls (C), the tumor-bearing mice in the TB group had markedly lower body weights (Figure 2a), higher mean organ weights (swollen lung, liver, and spleen), as well as lower weights of gastrocnemius muscle and adipose tissue (white and brown fat) (Figure 2b). Compared to the TB group, tumor-bearing mice receiving Se/FO (TB-N group) had markedly higher body weights, smaller tumor sizes and weights, lower organ weights, and higher weights of gastrocnemius muscle and adipose tissue. 

The tumor-bearing mice treated with gefitinib or erlotinib (TB-G and TB-E groups, respectively) had tumors of significantly smaller sizes and weights, higher body weights, lower organ weights, and higher muscle and adipose tissue weights than those of the untreated tumor-bearing mice. The mice treated with Se/FO in addition to gefitinib or erlotinib (TB-I-N and TB-T-N groups) exhibited a greater reduction in tumor sizes and weights than those treated with gefitinib or erlotinib alone. 

Tumors isolated from mice treated with and without nutritional supplements (Se/FO) differed significantly in size from those of the corresponding group (Figure 2c). Mice in the TB-N group had significantly fewer large liver and lung metastases than did those in the TB group, and mice in the TB-G-N and TB-E-N groups exhibited fewer small liver metastasis than did those in the TB-G and TB-E groups, respectively (Figure 2d). 

### 2.3. Effects of Combination Treatment on Survival and Serum IL-6 Levels of LLC1 Tumor-Bearing Mice

No significant difference in survival was observed among the six groups, with a survival rate of 100% in all groups (data not shown). Additionally, there were significantly lower serum interleukin (IL-6) levels in the TB-N, TB-G-N, and TB-E-N groups compared with the TB, TB-G, and TB-E groups, respectively (Figure 2e).

### 2.4. Effects of Combination Treatment on Tumor Transmembrane Receptors, β-Catenin, and GSK-3β Levels

**Figure 3:**
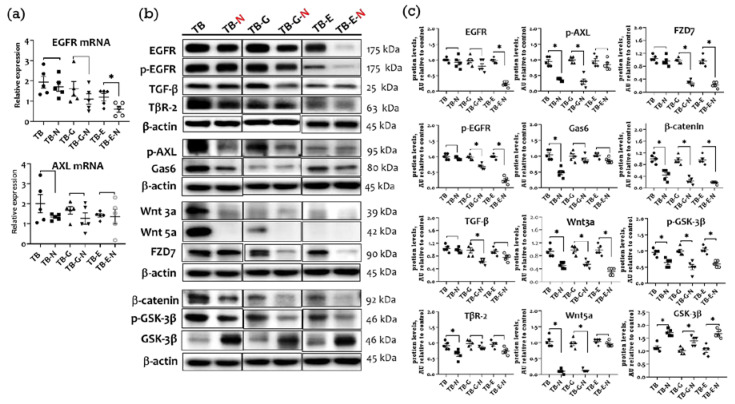
Expression of tumor receptor-signaling molecules in Lewis LLC1 tumor-bearing mice. (**a**) EGFR and AXL mRNA levels; (**b**) Protein expression of EGFR, TGF-β/TβR2, AXL/Gas6, Wnt3a/5a/FZD7, and β-catenin/GSK-3β; and (**c**) Densitometric analysis of Western blots. Quantitative values are expressed as the mean ± SEM of five independent samples in each group. Furthermore, the tumor homogenates pooled from 5 mice per group were loaded on each blot for the expression of target proteins. * *p* < 0.05 TB vs. TB-N, TB-G vs. TB-G-N, TB-E vs. TB-E-N. Treatment for each group is as in Figure 1.

Lower expression levels of EGFR mRNA were found in the TB-N, TB-G-N, and TB-E-N groups than in the TB, TB-G, and TB-E groups, respectively (Figure 3a). TB-E-N mice exhibited lower levels of EGFR mRNA than did those in the TB-G-N group. AXL receptor tyrosine kinase and its ligand growth arrest-specific 6 (Gas6) have been implicated in tumor growth and proliferation of NSCLC. The level of AXL mRNA was non-significantly lower in the TB-N and TB-G-N groups than in the TB and TB-G groups, respectively.

A trend toward lower protein levels of EGFR and phosphorylated EGFR (p-EGFR), transforming growth factor beta (TGF-β) and TGF-β receptor (TβR-2), p-AXL and Gas6, Wnt3a/5a and FZD7, β-catenin, and GSK-3β was showed in the TB-N, TB-G-N, and TB-E-N groups compared with the TB, TB-G, and TB-E groups, respectively (Figure 3b). Mice receiving FO/Se (TB-N group) had significantly lower levels of TβR-2, p-AXL/Gas6, Wnt3a/5a and FZD7, and β-catenin protein than did those in the TB group. Tumor-bearing mice in the TB-G-N group showed markedly lower levels of p-EGFR, TGF-β, p-AXL, Wnt3a, and β-catenin proteins compared to those in the TB-G group. Markedly lower expression of EGFR, p-EGFR, and β-catenin proteins was observed in the TB-E-N group than in the TB-E group. 

### 2.5. Effects of Combination Treatment on Expression of Tumor Angiogenic Markers

**Figure 4:**
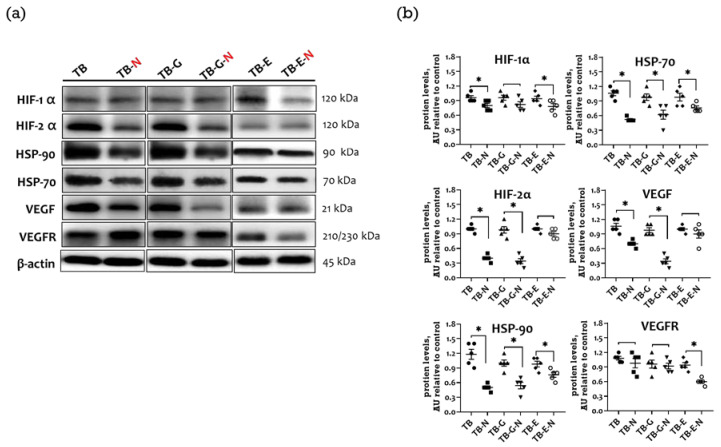
Tumor angiogenic marker expression in Lewis LLC1 tumor-bearing mice. (**a**) Protein expression of angiogenic markers; and (**b**) Densitometric analysis. Quantitative values are expressed as the mean ± SEM of five independent samples in each group. Furthermore, the tumor homogenates pooled from five mice per group were loaded on each blot for the expression of target proteins. * *p* < 0.05 TB vs. TB-N, TB-G vs. TB-G-N, TB-E vs. TB-E-N. Treatment for each group is as in Figure 1.

The upregulation of hypoxia-inducible factors (HIFs) and their chaperone, heat shock proteins (HSPs), increases the expression of the vascular endothelial growth factor (VEGF), which is involved in tumor angiogenesis and progression. Compared with the TB group, those mice in the TB-N group showed markedly reduced HIF-1α, HIF-2α, HSP-70, HSP-90, and VEGF protein expression levels (Figure 4a,b). Mice treated with both gefitinib and FO/Se (TB-G-N group) expressed significantly lower levels of HIF-2α, HSP-70, HSP-90, and VEGF protein than did those in the TB-G group. Mice treated with both erlotinib and FO/Se (TB-E-N group) expressed significantly lower levels of HIF-1α, HSP-70, HSP-90, and VEGF receptor (VEGFR) protein compared to those treated with erlotinib alone.

### 2.6. Effects of Combination Treatment on Tumor EMT Markers and Metastasis

**Figure 5:**
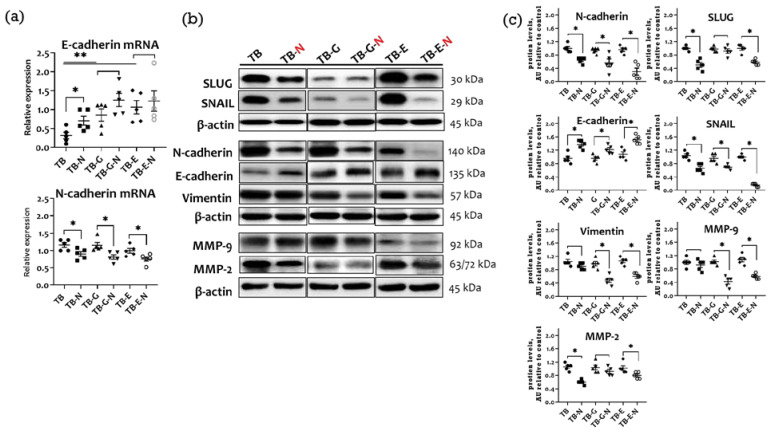
Comparison of epithelial to mesenchymal transition (EMT) markers and metastasis in Lewis LLC1 tumor-bearing mice according to treatment. (**a**) mRNA levels of E-cadherin and N-cadherin; (**b**) Protein expression of EMT and metastatic factors and MMPs; and (**c**) Densitometric analysis. Quantitative values are expressed as the mean ± SEM of five independent samples in each group. Furthermore, the tumor homogenates pooled from five mice per group were loaded on each blot for the expression of target proteins. In **a**, * *p* < 0.05 TB vs. TB-N, TB-E vs. TB-E-N, TB-G vs. TB-G-N, TB-E vs. TB-E-N. ** *p* < 0.05 TB vs. TB-G, TB-E. In **c**, * *p* < 0.05 TB vs. TB-N, TB-G vs. TB-G-N, TB-E vs. TB-E-N. Treatment for each group is as in Figure 1.

The activation of the EMT increases tumor invasiveness and metastatic activity. Tumor-bearing mice treated with Se/FO (TB-N group) had significantly higher tumor E-cadherin and lower N-cadherin mRNA levels than did those without Se/FO (Figure 5a). The TB-N mice also had markedly lower protein levels of matrix metalloproteinases (MMP-2 and MMP-9), mesenchymal markers (vimentin and N-cadherin), and EMT-activated transcription factors (SLUG and SNAIL), as well as a higher expression of E-cadherin protein than did those without Se/FO (Figure 5b,c).

Mice receiving gefitinib and Se/FO (TB-G-N group) had significantly lower N-cadherin mRNA and protein levels, lower protein levels of vimentin, MMP-2, MMP-9, and SNAIL, and higher protein levels of E-cadherin than did those treated with gefitinib alone (TB-G group). Compared with the mice treated with erlotinib alone (TB-E group), those treated with erlotinib and Se/FO (TB-E-N group) had markedly lower levels of N-cadherin mRNA, lower protein levels of vimentin, MMP-2, MMP-9, SLUG, and SNAIL, and higher levels of E-cadherin protein.

### 2.7. Effects of Combination Treatment on Immune Checkpoint Molecules Expression

**Figure 6:**
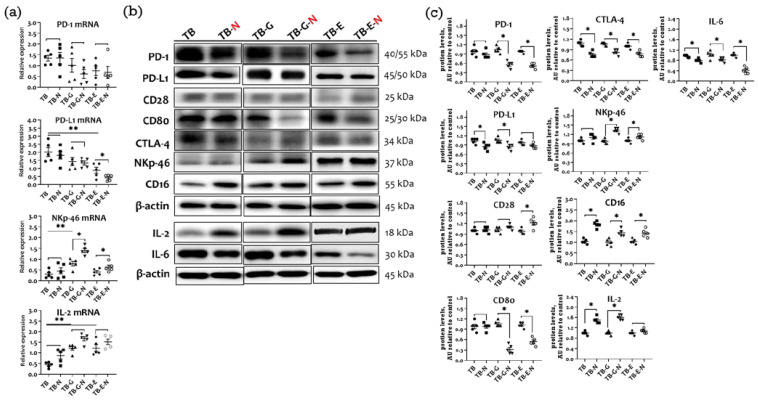
Tumor immune checkpoint molecules and cytokines in Lewis LLC1 tumor-bearing mice according to treatment. (**a**) mRNA levels of PD-1, PD-L1, NKp46, and IL-2; (**b**) Protein expression of immune checkpoint molecules and cytokines; and (**c**) Densitometric analysis. Quantitative values are expressed as the mean ± SEM of five independent samples in each group. Furthermore, the tumor homogenates pooled from five mice per group were loaded on each blot for the expression of target proteins. In (**a**), * *p* < 0.05 TB-E vs. TB-E-N, TB-G vs. TB-G-N, TB-E vs. TB-E-N. ** *p* < 0.05 TB vs. TB-G, TB-E. In (**c**), * *p* < 0.05 TB vs. TB-N, TB-G vs. TB-G-N, TB-E vs. TB-E-N. Treatment for each group is as in Figure 1.

The inhibitors of the immune checkpoint proteins programmed cell death ligand-1 (PD-L1), programmed cell death-1 (PD-1), and cytotoxic T lymphocyte-associated protein-4 (CTLA-4) are therapeutic agents used to treat NSCLC. The PD-1 mRNA level was markedly lower in mice treated with gefitinib and Se/FO (TB-G-N group) than in those treated with gefitinib alone (TB-G group) (Figure 6a). The tumors of mice in the TB-N group expressed higher mRNA levels of NKp46, CD16, and IL-2 that did those in the TB group. Furthermore, the TB-N group had markedly lower levels of PD-L1, PD-1, CTLA-4, and IL-6 and higher levels of NKp46, CD16, and IL-2 proteins compared with the TB group (Figure 6b,c). 

Compared with the untreated tumor-bearing mice, the gefitinib-treated mice in the TB-G group expressed markedly lower mRNA levels of NKp46 and CD16 and higher IL-2 mRNA levels (Figure 6a). The erlotinib treatment (TB-E group) also resulted in lower PD-L1 and IL-2 mRNA levels than in untreated tumor-bearing mice. Compared with mice treated with gefitinib alone (TB-G group), those treated with gefitinib and Se/FO (TB-G-N group) expressed dramatically higher levels of IL-2 protein and CD16 as well as NKp46 mRNA and protein, and lower levels of CD80, CTLA-4, PD-1, PD-L1, and IL-6 protein. Compared to mice treated with erlotinib alone (TB-E group), those treated with erlotinib and Se/FO (TB-E-N group) expressed markedly lower PD-L1 and higher NKp46 mRNA levels, had higher protein expressions of CD28, CD16, NKp46, and IL-2, and had lower protein expressions of CD80, CTLA-4, PD-1, and IL-6.

### 2.8. Effects of Combination Treatment on Tumor Proliferation, Cell Cycle Proteins, and Apoptosis

**Figure 7:**
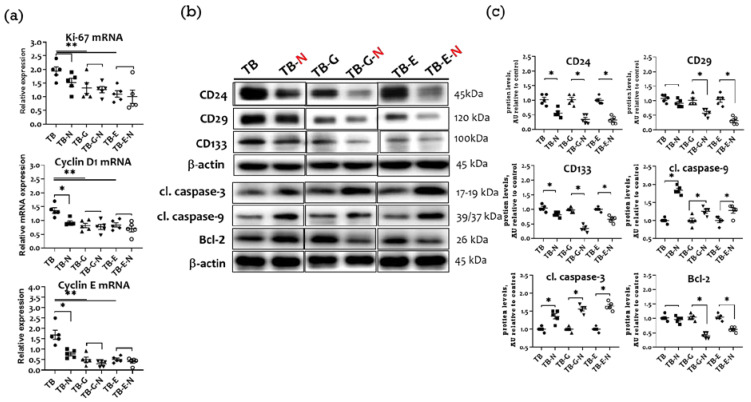
Tumor proliferation, cell cycle, cancer cell stemness, and apoptosis markers in Lewis LLC1 tumor-bearing mice according to treatment. (**a**) mRNA levels of Ki-67, cyclinD1, and cyclin E; (**b**) Protein expression of cancer stem cell and apoptotic markers; and (**c**) Densitometric analysis of Western blot. Quantitative values are expressed as the mean ± SEM of five independent samples in each group. Furthermore, the tumor homogenates pooled from five mice per group were loaded on each blot for the expression of target proteins. In **a**, * *p* < 0.05 TB vs. TB-N, ** *p* < 0.05 TB vs. TB-G, TB-E. In **c**, * *p* < 0.05 TB vs. TB-N, TB-G vs. TB-G-N, TB-E vs. TB-E-N. Treatment for each group is as in Figure 1.

Ki-67 is a cancer proliferation marker, and cyclins D1 and E are major regulators of cell cycle progression. Compared with untreated tumor-bearing mice, those treated with Se/FO alone (TB-N group) expressed significantly lower mRNA levels of Ki-67, cyclin D1, and cyclin E (Figure 7a), markedly higher cleavage levels of apoptosis-related proteins caspase-3 and caspase-9, and non-significantly lower protein expressions of the cancer stem cell (CSC) markers CD24, CD29, and CD133 (Figure 7b,c). 

The mice treated with gefitinib and Se/FO (TB-G-N group) expressed non-significantly lower mRNA levels of Ki-67, cyclin D1, and cyclin E than those treated with gefitinib alone (TB-G group), had significantly lower expressions of CD24, CD29, CD133, as well as a higher cleavage of caspase-3 and caspase-9 and a lower anti-apoptotic Bcl-2 protein expression (Figure 7b,c). Similarly, mice treated with erlotinib and Se/FO in the TB-E-N group expressed non-significantly lower mRNA levels of Ki-67, cyclin D1, and cyclin E, significantly lower levels of CSC markers, higher cleavage levels of caspase-3 and caspase-9, and lower Bcl-2 proteins.

## 3. Discussion

This preliminary study compares the anticancer efficacy of EGFR-TKIs (gefitinib or erlotinib) in Lewis LLC1 tumor-bearing mice when administered alone or in combination with Se and FO. We observed that the combination treatment with Se/FO and either of the EGFR-TKIs resulted in tumors with lower weights and smaller sizes, lower metastases, and higher body masses of muscle and fat compared to those treated with the EGFR-TKI alone. Nutritional supplementation with Se/FO could serve as a potential modulator to improve the treatment efficacy of first-generation EGFR-TKI by regulating multiple targets in a non-EGFR mutant NSCLC tumor model. 

Our results show that combined EGFR inhibitors and Se/FO treatment suppress EGFR expression in tumors more than EGFR inhibitor therapy alone. Docosahexaenoic acid (DHA) and eicosapentaenoic acid (EPA) are the major n-3 poly-unsaturated fatty acids present in the FO component. DHA and EPA may be potential EGFR antagonists, thus reducing the activation of the EGFR signaling pathway [27]. DHA decreased the cell viability in H1299, and KRAS-mutant A549 and LLC1 lung cancer cells in a dose-dependent manner, via the EGFR and downstream proteins’ inhibition [28,29]. The combined DHA and gefitinib treatment suppressed the EGFR signaling in EGFR-mutant human NSCLC PC9 and TKI-resistant A549 lung cancer cells [30]. The combined treatment with Se and radiation is more effective and results in the markedly improved inhibition of EGFR expression in human lung cancer cells (NCI-H460 and H1299) without EGFR mutation than in the treatment with radiation alone [31]. Additionally, the combination treatment with Se/FO and chemotherapeutic agents increases the efficacy of EGFR inhibition in the tumor tissues of mammary tumor-bearing mice [27]. 

The present study further demonstrates that the combination treatment modulates the expression of the tumor receptor signaling molecules, including TGF-β/TβR-2, AXL/Gas6, and Wnt/FZD/β-catenin/GSK-3β, in LLC1 tumor tissues. Recent studies report that the blockade of TGF-β/TβR, Wnt/β-catenin, and AXL/Gas6 signaling can potentially reduce the EGFR-TKI resistance in lung cancer A549, HCC827, and PC9 cell lines [3,4,11]. Treatments with FO result in a significant reduction in the serum levels of TGF-β in the bladder cancer model [32]. The combination treatment with FO and Se suppresses AXL expression in HCC827 lung adenocarcinoma cells [19], although few studies show either FO or Se modulates AXL/Gas6 expression in cancer cells. Our previous studies have shown that the Se/FO combination treatment increases the therapeutic efficacy of anti-cancer agents against breast cancer via the dose-dependent downregulation of TGF-β/TβR-2, AXL/Gas6, and β-catenin signaling [27]. Se suppresses the growth of HT-29 colorectal cancer cells by inhibiting Wnt/β-catenin signaling [33]. The downregulation of Wnt/β-catenin signaling through DHA/EPA treatment is also linked to growth inhibition in human pancreatic cancer cells [34]. Thus, the combination treatment in Lewis LLC1-tumor-bearing mice regulates these receptor kinase signaling pathways.

Angiogenesis is an important prognostic factor in advanced NSCLC. HIF-1α/HIF-2α regulates multiple genes involved in the response to hypoxia, promoting angiogenesis and EMT activation in NSCLC [35,36]. HSP-70 and HSP-90 are also highly expressed in tumors and contribute to high tumor invasion and cancer stemness [37]. Studies have shown that EPA and DHA have potent anti-angiogenic effects on cancer cells via the inhibition of HIF-1α, VEGF, and VEGFR production [38,39,40]. Additionally, Se inhibits the activity of HIF-1α, HIF-2α, and VEGF in cancer cells [41]. The present study shows that Se/FO increases the erlotinib-induced reduction in the expression of HIF-1α and VEGFR and increases the gefitinib-induced reduction in HIF-2α and VEGF expression in tumors. These combinations also result in even greater reductions in the levels of HSP-70/HSP-90. 

In addition, the present study demonstrates that combination treatment has greater inhibitory effects on metastases and invasiveness than EGFR inhibitor therapy alone. The activation of the EMT upregulates the expression of EMT-related transcription factors and mesenchymal markers through TGF-β and Wnt signaling, MMPs, and HIF-1α, increasing the invasiveness of cancer cells [42]. Erlotinib suppressed the TGF-β1–induced EMT phenotype in A549 cells [43] and inhibited the MMP-9 levels in the LLC1 cells and LLC1 tumor-bearing mice [21]. A recent study showed that DHA inhibits EMT-related markers and invasion by inhibiting TGF-β in colorectal carcinoma cells [44]. 

CTLA4 competes with CD28 receptors to bind to CD80, thereby inhibiting T-lymphocyte activation [45], but EPA treatment suppressed CD4+ T-cell activation by inhibiting CTLA4, with no change in CD28 expression [46]. In EGFR-mutant NSCLC cells, PD-L1 upregulates TGF-β signaling to activate the EMT pathway, thereby contributing to acquired resistance to gefitinib [47]. Additionally, AXL inhibition increases the efficacy of anti-PD-1 therapy in mutant KRAS-driven NSCLCs [48]. Higher circulating IL-6 levels are associated with immunotherapeutic resistance. The combined inhibitors of IL-6 and CTLA-4, by contrast, improve the survival of LLC1 tumor-bearing mice [49]. Recent studies showed that Se treatment suppressed cancer cell survival and induced apoptosis in vitro through the blockade of PD-L1 [15]. Se also ameliorated NK cell activation and cytotoxicity attributed to the upregulation of IL-2 receptors on the surface of NK cells [50]. Our results show that combined treatment decreases the levels of PD-1, PD-L1, CTLA-4, CD80, and IL-6 and increases the expression of surface receptors on NK cells and IL-2 in Lewis LLC1 tumors. 

The expression of the high proliferation marker Ki-67 is an indicator of poor prognoses in NSCLC patients [51]. Erlotinib increased the sub-G1 population of lung cancer cells, while gefitinib reduced this population of lung cancer cells [52]. Supplemental FO intake decreased Ki-67 levels in benign hyperplastic breast tissue [53]. DHA and EPA triggered cell cycle arrest at the G0/G1 phase, which was accompanied by a reduction in the protein levels of CDK2 and cyclin E in human cancer cells [54]. The present results show that mice receiving either supplemental FO/Se or an EGFR inhibitor have significantly lower expression of Ki67 and the cell-cycle marker proteins cyclin D1/E than untreated tumor-bearing mice. 

Several surface markers for lung cancer stemness have been identified, including CD133, CD29, and CD24. EPA treatment decreases CD133 and increases the sensitivity to colorectal cancer chemotherapy [55]. The combined treatment with Se and FO resulted in the greater suppression of CD44 and CD133 expression than FO alone in gefitinib-resistant HCC827 cells [19]. We observed here that the combination treatment in LLC1 tumor-bearing mice markedly reduced the expression of NSCLC stemness markers. Thus, Se plus FO may alter the CSC phenotype that contributes to CSC inhibition.

Gefitinib and erlotinib inhibit the activation of EGFR-mediated PI3K/Akt/mTOR in A549, A549-gefitinib-resistant, KRAS-mutant H358, and H441 cells, leading to lung cancer cell apoptosis [56,57]. Positive crosstalk between the TβR, AXL, Wnt/β-catenin, and EGFR can contribute to the activation of PI3K/Akt/mTOR signaling, blocking apoptotic pathways in NSCLC [3,7,58]. Our results indicate that supplemental Se/FO downregulates EGFR/TβR/AXL/Wnt/β-catenin and increases apoptotic signaling induced by EGFR-TKI. Se supplementation triggers the phosphorylation of Bcl-2 and apoptosis of neuroblastoma cells under hypoxia [59]. EPA and DHA increase the apoptosis of NSCLC A549 and A427 cells linked to Akt inactivation [60]. Previous studies have also reported that Se/FO supplements target EGFR/TβR/AXL/PI3K/Akt/mTOR signaling and therefore increase the apoptotic efficacy of anti-cancer agents in breast-cancer-bearing mice and NSCLC cells [17,27]. 

## 4. Materials and Methods

### 4.1. Anti-Cancer Agents and Nutritional Supplements

Gefitinib (Iressa, AstraZeneca, UK) and erlotinib (Tarceva, OSI Pharmaceuticals, Melville, NY, USA) were purchased from commercial sources. The nutritional supplements FO and Se yeast were premixed with a control powder (Do Well Laboratories, Irvine, CA, USA) as described previously [13,27]. The final concentrations of FO and elemental Se were 6.7 mg/g and 1.5 µg/g, respectively. The other chemical reagents were of analytical grade and were obtained from commercial suppliers unless stated otherwise.

### 4.2. Cell Culture and Animal Experiments

Murine Lewis lung carcinoma cells (LLC1)(ATCC CRL-1642) were obtained from the Bioresource Collection and Research Center (BCRC, Hsinchu, Taiwan). The cells were cultured in Dulbecco’s modified Eagle’s medium (DMEM), powdered high-glucose supplemented with 10% heat-inactivated fetal bovine serum (Thermo Fisher Scientific, Waltham, MA, USA), 2 mmol/L L-glutamine, 100 mg/mL streptomycin, and 100 U/mL penicillin in a humified incubator containing 5% CO_2_ at 37 °C. 

MTT assay was used to determine the viability of LLC1 cells. Briefly, cells were seeded at a density of 1 × 10^4^ cells/well in 96-well plates and then incubated with different levels of gefitinib (0, 2, 4, 8, 16, and 32 μM) or erlotinib (0, 10, 20, 40, 50, and 60 μM) for 48 h. After the incubation period, 20 µL of MTT was added to all wells and further incubated at 37 °C for 3 h. The optical density value was then evaluated at 570 nm. Viability was expressed as a percentage of optical density in treated cells relative to that in control cells.

Animal experiments were approved by the Institutional Animal Care Committee of Hung Kuang University. Six-week-old healthy C57BL/6 male mice were obtained from the National Laboratory Animal Centre (Nangang Taipei, Taiwan). Mice were housed in a controlled environment with a 12 h light/dark period at 24 ± 1 °C and 60–70% relative humidity. A one-week acclimatization period was allowed for the animals, and rodent chow (Lab Diet #5001, Ralston Purina, St. Louis, MO, USA) and distilled deionized water were made available ad libitum to all animals throughout the experimental period.

A Lewis lung carcinoma mouse model was established according to recent studies [21,22,23], as described below. On day 0 of the experiment, LLC1 cells (1 × 10^6^) in 100 µL normal saline were subcutaneously injected into the right hind thigh of the mice. On day 6, tumor-bearing mice were randomized into 6 weight-matched groups of 5 mice each, as follows: (1) TB and TB-N groups: mice receiving either normal saline (TB) or nutritional supplement FO/Se (TB-N); (2) TB-G and TB-G-N groups: mice treated with gefitinib (50 mg/kg/day by oral gavage on days 6–16) alone (TB-G) or in combination with FO/Se (0.5 g, by oral gavage twice daily on days 6–20) (TB-G-N); (3) TB-E and TB-E-N groups: mice treated with erlotinib (10 mg/kg/day by oral gavage on days 6–16) alone (TB-E) or in combination with FO/Se (0.5 g, by oral gavage twice, 8 hr apart, a day from day 6 to day 20) (TB-E-N). Additionally, healthy controls were allocated to C and C-N groups according to treatment with either normal saline or FO/Se, respectively.

All mice were euthanized after blood collection on Day 21 since cancer survival was not the primary purpose of this investigation. Primary tumors, lungs, livers, spleens, and brains were carefully excised and weighed. Tumor volume was calculated using the formula [(short diameter in mm)^2^ × (long diameter in mm)]/2 based on manual caliper measurements. Furthermore, the determination of metastasis in a distal organ’s surface was determined by visual inspection and classification. Small metastases were defined as tumor nodules < 2 mm in the lung tissue, and large metastases were defined as those > 0.5–1 cm. 

### 4.3. Determination of Serum IL-6

Serum levels of mouse IL-6 were assayed according to the instructions of Quantikine ELISA IL-6 immunoassay kits (M6000B, R&D Systems, Inc., Minneapolis, MN, USA). In brief, each sample was added to the individual well and incubated for 2 h at room temperature. Then 100 μL of mouse IL-6 conjugate was added to each well and it was incubated for 2 h, followed by repeated washing. Finally, we added a stop solution to each well. Absorbance values of each well were detected at 450 nm with the correction wavelength set at 540 nm or 570 nm.

### 4.4. Western Blot Analysis

Parts of the tumor specimen were extracted in a homogenization buffer containing 1% NP-40, 0.5% sodium deoxycholic acid, and 0.1% SDS, supplemented with a protease inhibitor cocktail (Roche Diagnostics GmbH, Mannheim, Germany) [24]. The total protein concentrations of lysates were measured by Bio-Rad Protein Assay (Bio-Rad, Hercules, CA, USA) using a series of bovine serum albumin as standards. A total of 50 μg per sample was separated on a homemade 10 or 12% SDS-polyacrylamide gel electrophoresis and then transferred onto nitrocellulose membranes and incubated with different primary antibodies (Table S1) overnight at 4 °C, followed by incubation with horseradish peroxidase-conjugated rabbit (#7074, Cell signaling, Danvers, MA, USA), goat (sc-2354, Santa Cruz, Dallas, TX, USA), and anti-mouse secondary antibodies for one hour at room temperature. The blots were then visualized using an enhanced chemiluminescence detection kit (PerkinElmer Life Sciences Inc. Waltham, MA, USA). Anti-β-actin monoclonal was used as a loading control. Finally, signal intensities were measured using the Luminescent image system (FUJIFILM, LAS-4000, Tokyo, Japan) and Multi Gauge 3.0 software (Fuji, Japan). Densitometry analysis of each bot was normalized by β-actin (*n* = 5 mice were analyzed in each group).

### 4.5. RNA Isolation and Real-Time qPCR Analysis

Total RNAs from tumors were extracted using the Bio-Rad RNA kit (Bio-Rad Lab, Hercules, CA, USA) and RNAs were then used for cDNA synthesis using a thermocycler (T100 Thermal Cycler, Bio-Rad, Hercules, CA, USA) with the iScript cDNA synthesis kit (Bio-Rad Lab, Hercules, CA, USA) according to the manufacturer instructions. Briefly, amplified cDNA was assessed by the CFX Connect RT-PCR detection system using the SYBR Green Supermix (Bio-Rad Lab, Hercules, CA, USA) and was normalized to 18S as the housekeeping gene. Fold changes between samples were measured using the 2-ΔΔCt method. *N* = 5 mice were analyzed in each group. The primer pair sequences for quantitative real-time PCR used were list in Table S2.

### 4.6. Statistical Analysis

Continuous variables were presented as the mean (standard error of the mean, SEM). The normality of the measurements was determined by using the Shapiro–Wilk test. Student’s t-test and one-way ANOVA (analysis of variance) followed by post hoc analysis with Duncan’s multiple-range tests were used for comparisons as appropriate. A two-tailed *p*-value less than 0.05 was considered statistically significant.

## 5. Conclusions

In summary, our preliminary results show that combination therapy using Se/FO differentially enhances the responses of Lewis LLC1 tumor-bearing mice to treatments with gefitinib or erlotinib via the modulation of the receptor signaling and immune checkpoint molecules. This combination treatment further results in the greater inhibition of angiogenesis and cancer stemness, the EMT, metastases, as well as the proliferation and cell cycle, and increases apoptosis in tumor tissues. Thus, supplemental Se plus FO can offer a therapeutic alternative to gefitinib or erlotinib therapy for Lewis lung carcinoma. Future experiments will be needed to clarify the therapeutic effects of combination treatment in other KRAS mutant mouse models of NSCLC. Moreover, we found that Se/FO markedly reduces tumors’ HSP-70/-90 protein levels, and consequently may promote the efficacy of hyperthermia treatments and act as a chemosensitizer in advanced NSCLC therapy. A further study is underway to evaluate the anti-cancer efficacy of a combination treatment with hyperthermia and Se/FO in an NSCLC model.

## Data Availability

All data generated or analyzed during this study are included in this manuscript.

## Supplementary Materials

The following supporting information can be downloaded at: https://www.mdpi.com/article/10.3390/md20120751/s1, Table S1. List of antibodies used in this study. Table S2. The primer pair sequences for quantitative real-time PCR used in this study.
