# Supplementary material for: Targeting EGFR in Combination with Nutritional Supplements on Antitumor Efficacy in a Lung Cancer Mouse Model
Source: Mar Drugs. 2022 Nov 29;20(12):751. doi: 10.3390/md20120751 (PMC9783964; doi:10.3390/md20120751)
Supplement: Supplementary file 1 [file marinedrugs-20-00751-s001.zip › marinedrugs-2032981-supplementary.pdf]

Table S1. List of antibodies used in this study.

EGFR (1:1000, ab52894, Abcam)	N-cadherin (1:1000, 13116, Cell signaling)
p-tyr1068-EGFR (1:1000, #2234, Cell signaling)	E-cadherin (1:1000, #3195, Cell signaling)
TGF-β (1:1000, #3711, Cell signaling)	Vimentin (1:1000, #5741, Cell signaling)
TGF-βR2 (1:1000, ab61213, Abcam)	SNAIL (1:1000, #3879, Cell signaling)
p-tyr698/tyr702/tyr703-AXL (1:1000, orb4400, Biorbyt)	SLUG (1:1000, #9585, Cell signaling)
Gas6 (1:1000, GTX31628, Gene Tex)	cl-caspase-3 (1:1000, #9662, Cell signaling)
HIF-1α (1:500, sc-13515, Santa Cruz)	cl-caspase-9 (1:1000, #9508, Cell signaling)
HIF-2α (1:1000, ab109616, Abcam)	Bcl-2 (1:5000, sc-16323, Santa Cruz)
Hsp70 (1:1000, #4872, Cell signaling)	Gas6 (1:1000, GTX31628, GeneTex)
Hsp90α/β (1:1000, sc-13119, Cell signaling)	Wnt3a (1:1000, sc136163, Santa Cruz)
VEGF (1:1000, sc-7269, Santa Cruz)	Wnt5a (1:1000, GTX111187, Gene Tex)
VEGFR2 (1:1000, #2479, Cell signaling)	FZD7 (1:1000, ab64636, Abcam)
MMP-2 (1:500, sc-53630, Santa Cruz)	β-catenin (1:1000, sc7963, Santa Cruz)
MMP-9 (1:500, sc-13520, Santa Cruz)	GSK3β (1:1000, #9832, Cell signaling)
p-Ser9-GSK3β (1:1000, #5558, Cell signaling)	CTLA-4 (1:1000, sc-376016, Santa Cruz)
CD24 (1:1000, #2514189, Millipore)	NKp46 (1:1000,orb336679, biorbyt)
CD29 (1:2000, ab179471, Abcam)	CD16 (1:1000, E-AB-70305, Elabscience)
CD133 (1:750, E-AB-33462, Elabscience)	PD-1 (1:1000, 18106-1-AP, Proteintech)
CD28 (1:1000, ab205136, Abcam)	PD-L1 (1:1000, 66248-lg, Proteintech)
CD80 (1:500, ab215166, Abcam)	IL-2 (1:1000, 60306-1-lg, Proteintech)
β-actin (1:1000, #8457, Cell signaling)	IL-6 (1:1000, 21865-1-AP, Protein-tech)

Table S2. The primer pair sequences for quantitative real-time PCR used in this study.

EGFR	forward, TTGACGGAAGGGCACCACCAG; reverse, TCAAAGGCCTCACGA TCACC
Ki-67	forward, CTGCCTGCGAAGAGAGCATC; reverse, AGCTCCACTTCGCCTTT TGG
NKp46	forward, GCACATCCTTCCTCCTA; reverse, CCTCTGGCTC TTACGAT
AXL	forward, GGTGTTTGAGCCAACCGTGGAA; reverse, GCCACTTATGCCG ATCTACCA
IL-2	forward, TGTGTTGTAAGCAGGAGGTACA; reverse, GATGGATA GCCTT CTGTCAAAGC
PD-1	forward, TTCAGGTTTACCACAAGTGG; reverse, TGACAATAGGAAACC GGAA
PD-L1	forward, GAAGTCCAGCTCCTCA TA; reverse, TTCTGCTCAACAAGTATG ATGTC
Cyclin D1	forward, CTGGCGCA GGCTTGACTC; reverse, CATCAAGTGTGACCCGG ACT
Cyclin E	forward, CAGC TTGGATTTGCTGGACAAAG; reverse, TGTCAGGACCACA CTCGGA
N-cadherin	forward, GTTCTCCACTTGATTGCCATTGA; reverse, GATCGAGAGCTGAT AGCCC G
E-cadherin	MP200650, Sino Biological Inc.; Tag Sequence: GATTACAAGGATG ACGACGATAAG; Sequencing Primers: T7 (TAATACGACTCACTATAGGG) and BGH (TAGAAGGCACAGTCGAGG)
18S	forward, TTGACGGAAGGGCACCACCA G; reverse, TCAAAGGCCTCACG ATCACC
